# Estimating prevalence and test accuracy in disease ecology: How Bayesian latent class analysis can boost or bias imperfect test results

**DOI:** 10.1002/ece3.6448

**Published:** 2020-06-15

**Authors:** Sarah K. Helman, Riley O. Mummah, Katelyn M. Gostic, Michael G. Buhnerkempe, Katherine C. Prager, James O. Lloyd‐Smith

**Affiliations:** ^1^ Department of Ecology and Evolutionary Biology University of California, Los Angeles Los Angeles CA USA; ^2^ Department of Internal Medicine Southern Illinois University School of Medicine Springfield IL USA; ^3^ Fogarty International Center National Institutes of Health Bethesda MD USA

**Keywords:** Bayesian latent class, California sea lion, diagnostic test, disease, infection, prevalence, sensitivity, specificity

## Abstract

1. Obtaining accurate estimates of disease prevalence is crucial for the monitoring and management of wildlife populations but can be difficult if different diagnostic tests yield conflicting results and if the accuracy of each diagnostic test is unknown. Bayesian latent class analysis (BLCA) modeling offers a potential solution, providing estimates of prevalence levels and diagnostic test accuracy under the realistic assumption that no diagnostic test is perfect.
2. In typical applications of this approach, the specificity of one test is fixed at or close to 100%, allowing the model to simultaneously estimate the sensitivity and specificity of all other tests, in addition to infection prevalence. In wildlife systems, a test with near‐perfect specificity is not always available, so we simulated data to investigate how decreasing this fixed specificity value affects the accuracy of model estimates.
3. We used simulations to explore how the trade‐off between diagnostic test specificity and sensitivity impacts prevalence estimates and found that directional biases depend on pathogen prevalence. Both the precision and accuracy of results depend on the sample size, the diagnostic tests used, and the true infection prevalence, so these factors should be considered when applying BLCA to estimate disease prevalence and diagnostic test accuracy in wildlife systems. A wildlife disease case study, focusing on leptospirosis in California sea lions, demonstrated the potential for Bayesian latent class methods to provide reliable estimates under real‐world conditions.
4. We delineate conditions under which BLCA improves upon the results from a single diagnostic across a range of prevalence levels and sample sizes, demonstrating when this method is preferable for disease ecologists working in a wide variety of pathogen systems.

## Graphical abstract

Obtaining accurate estimates of disease prevalence is crucial in disease ecology and wildlife population management, but can be difficult if different diagnostic tests yield conflicting results and if the accuracy of each diagnostic test is unknown. Bayesian latent class analysis (BLCA) provides estimates of prevalence levels and diagnostic test accuracy under the realistic assumption that no diagnostic test is perfect. Using simulations and a wildlife case study, we assess how the precision and accuracy of this method are affected by sample sizes, diagnostic test accuracy, and the choice of diagnostic threshold for quantitative assays, and further explore when the method improves upon the results from a single diagnostic test.

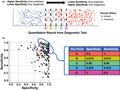

## 1 INTRODUCTION

Infection prevalence, or the fraction of individuals in a population that are infected with a pathogen at a given time, is a crucial metric of pathogen dynamics within ecological systems (Buhnerkempe et al., 2015; Haydon, Cleaveland, Taylor, & Laurenson, 2002; Viana et al., 2014). Knowledge of infection prevalence can elucidate disease dynamics in a system, providing data to health professionals aiming to mitigate disease risk and to scientists seeking to understand key mechanisms. The true infection prevalence within an ecological system is usually impossible to measure exactly but can be estimated by testing representative subsets of a population. However, it can be difficult to obtain large representative data sets to estimate disease prevalence in wildlife populations. Limitations including funding, personnel, regulatory restrictions, and the availability of tests appropriate to a specific study species typically determine which diagnostic tests can be used in a given wildlife system and how many individuals can be tested. Wildlife studies face additional challenges, as they are often restricted to the use of diagnostic tests whose accuracy may not be known if the tests have been validated in domestic animals, rather than the host species of interest (Moreno‐Torres, Wolfe, Saville, & Garabed, 2016).

While diagnostic test accuracy is sometimes overlooked in favor of more immediate challenges such as obtaining representative samples, it can have substantial impacts on disease prevalence estimates. Diagnostic tests vary in their sensitivity (probability of detecting true positives) and specificity (probability of detecting true negatives), so both individual diagnostic results and population‐level prevalence estimates will vary depending on the tests used in a given system. Furthermore, a set of imperfect diagnostic tests may show conflicting results in the same individual (e.g., due to differences in test accuracy or what disease state the tests are measuring). Assessing the true infection status of individuals from imperfect information and using this information to estimate population prevalence is a challenge facing epidemiologists and disease ecologists worldwide.

To complicate matters further, when considering a test with continuous quantitative output, users must choose a diagnostic threshold that separates negative test results from positive results. A trade‐off exists between sensitivity and specificity, such that this threshold can be lowered to make the test more sensitive (limiting the number of false‐negative results) or raised to make the test more specific (limiting the number of false‐positive results; Florkowski, 2008). Many tests that are conventionally viewed as binary, such as serology or even polymerase chain reaction (PCR), actually have underlying quantitative thresholds that could be tuned to maximize sensitivity or specificity, but not both. Disease ecologists and epidemiologists routinely use different thresholds for diagnostic assays, depending on their research aims and system characteristics (Almberg, Cross, Dobson, Smith, & Hudson, 2012).

In situations where careful choice of diagnostic threshold is not itself sufficient to improve prevalence estimates, a statistical method called Bayesian latent class analysis (BLCA) has been applied to facilitate estimates of infection prevalence and diagnostic test accuracy (Gonçalves et al., 2012; Limmathurotsakul et al., 2012; Muma et al., 2007; Pan‐ngum et al., 2013). When applying this technique, an individual's true clinical infection status is assumed to be a latent unobserved process. BLCA does not explicitly categorize each individual as infected or uninfected. Rather, each tested individual has a probability of being infected or uninfected, given their observed combination of test outcomes and the accuracy of each test. The model integrates probabilistic information about the true infection status of all tested individuals to simultaneously estimate overall infection prevalence, along with the sensitivity and specificity of each test, under the realistic assumption that no diagnostic test is perfect (Rindskopf & Rindskopf, 1986). Traditionally, BLCA methods assume conditional independence of test results, given the disease status of a tested individual. Recent research has addressed the issue of identifiability and potential for biases due to the underlying dependence structure among test results, as well as approaches to modeling conditional dependence and adding random effects to address these challenges (Albert & Dodd, 2004; Dendukuri & Joseph, 2001; Hadgu & Qu, 1998; Jones, Johnson, Hanson, & Christensen, 2010; Pepe & Janes, 2006; Qu, Tan, & Kutner, 1996). Since higher‐order information (e.g., longitudinal sampling) is unlikely to be available for diagnostic tests in wildlife hosts, here we analyze the performance of BLCA under the assumption of conditional independence (Wang & Hanson, 2019). This assumption is reasonable when diagnostic tests measure distinct biological processes that are not expected to be substantially correlated (e.g., the presence of a pathogen in urine vs the antibody response to a pathogen in the bloodstream; Kostoulas et al., 2017), and this study assesses the application of BLCA in systems where this assumption is valid.

Bayesian latent class analysis has been used primarily to estimate disease prevalence and test accuracy in domestic animals (Basso et al., 2013; Boelaert, Aoun, Liinev, Goetghebeur, & Van der Stuyft, 1999; Hartnack et al., 2013; Mathevon, Foucras, Falguières, & Corbiere, 2017; Muma et al., 2007; Nielsen, Toft, & Ersbøll, 2004) or humans (Gonçalves et al., 2012; Limmathurotsakul et al., 2012; Pan‐ngum et al., 2013; Schumacher et al., 2016), but it has also been applied sparsely in wildlife systems (Bronsvoort et al., 2008; Moreno‐Torres et al., 2016; Verma‐Kumar et al., 2012). The limitations and biases from test sensitivity and specificity, and situations where BLCA improves upon single test estimates, have not previously been explored in the context of wildlife. Our study assesses the accuracy and potential for bias across a range of biologically realistic sample sizes and prevalence levels by applying BLCA to simulated data. When using BLCA models, the specificity of the most accurate test is typically fixed at or close to 100% (Hartnack et al., 2013; Limmathurotsakul et al., 2012; Mathevon et al., 2017; Pan‐ngum et al., 2013; Schumacher et al., 2016), which is often not the case in real‐world conditions, particularly when dealing with wildlife. Our analysis relaxes this assumption, simulating diagnostic test data using multiple diagnostic test ensembles to investigate BLCA efficacy as fixed test specificity decreases from 100% to 80%. In doing so, we also provide actionable guidance for situations where the investigators can choose the diagnostic threshold to tune the specificity of their fixed test.

To demonstrate the application of this method in a wildlife system, we apply BLCA to *Leptospira* surveillance data from California sea lions (*Zalophus californianus*). The bacteria *Leptospira interrogans* serovar Pomona is one of the primary causes of strandings in this species, having caused cyclical outbreaks since the mid‐1980s that are associated with high morbidity and mortality (Greig, Gulland, & Kreuder, 2005; Lloyd‐Smith et al., 2007; Prager et al., 2013). Animals with the disease, known as leptospirosis, present with clinical signs associated with *Leptospira‐*induced kidney dysfunction (Cameron et al., 2008). While detection of *Leptospira* DNA in the urinary tract (Polymerase Chain Reaction ‐ PCR) is the definitive diagnosis, obtaining samples to test via PCR is often impossible, so high antibody titers (Microscopic Agglutination Test ‐ MAT) and serum chemistry markers indicative of *Leptospira*‐induced kidney dysfunction are also utilized to detect clinical infections. We used BLCA to estimate the prevalence of clinical infections in stranded California sea lions, along with test sensitivity and specificity, using results from these three diagnostic tests. We then simulated data using the parameter estimates from the sea lion data to assess BLCA estimates for prevalence and test accuracy under real‐world sample sizes and testing conditions. Finally, we compared prevalence estimates from the BLCA model to what would be estimated from a single diagnostic test, to understand the circumstances under which the BLCA method is most worthwhile. In combination, analyses of the simulated data and results of the wildlife case study provide insights into the use and limitations of BLCA in disease ecology.

## 2 METHODS

**FIGURE 1:**
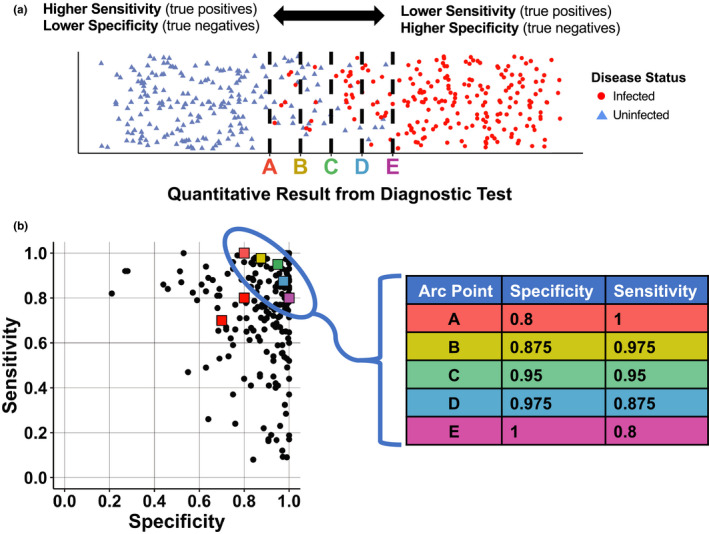
Infection status for a group of individuals relative to the sensitivity and specificity of test thresholds (top), and the values we chose for simulations relative to levels reported in the literature. (a) The true infection status (red circles = infected, blue triangles = uninfected) is plotted for hypothetical test results in a group of individuals. The *x*‐axis represents a range of quantitative test results, with lower test results on the left and higher test results on the right. A threshold must be chosen, above which value a test result is considered positive. Thresholds that correspond to points A‐E in b are shown as dashed black lines, demonstrating the trade‐off between sensitivity (True Positives/(True Positives + False Negatives)) and specificity (True Negatives/(True Negatives + False Positives)). (b) Diagnostic test sensitivities and specificities previously reported in the literature (Alberg et al. 2004; Maxim et al. 2014), shown as black circles. For data simulations, the sensitivity/specificity values of test 1 and test 2 were set at 70%/70% and 80%/80%, respectively (shown as red squares). The fixed specificity provided to the Bayesian model was selected from points A‐E (table on right)

The Bayesian latent class model used in this analysis requires binary test outcomes. Thus, tests that yield results on a continuous scale (e.g., serological titers or quantitative PCR Ct values) must be classified as positive or negative, based on whether the test result falls above or below a diagnostic threshold. This classification threshold can be selected to maximize sensitivity (Se) or specificity (Sp) and must be chosen carefully for each test (Figure 1a). We simulated results from three diagnostic tests, using a hypothetical diagnostic test ensemble by selecting pairs of sensitivities and specificities from a range of previously reported values for 193 medical tests in the literature (Alberg, Park, Hager, Brock, & Diener‐West, 2004; Maxim, Niebo, & Utell, 2014; Figure 1b). Two of the tests (tests 1 and 2) were assigned lower Se/Sp combinations (Figure S1b,c), representative of more mediocre diagnostic tests reported in the literature. The remaining test (test 3) in the ensemble was assigned properties chosen across an arc of Se/Sp values from 100%/80% to 80%/100% (Figure 1b: points A‐E), which corresponded to the highest Se/Sp combination of the three tests. This range was chosen because nearly one third of tests in the literature survey (*n* = 63/193) had both sensitivity and specificity > 80%, so we assumed biologists would usually have at least one diagnostic test that fell within this range. In each simulation, the specificity of test 3 (which was always the highest specificity of all three tests) was fixed in the BLCA model.

### 2.1 Parameter selection

We chose a range of biologically realistic parameter sets, using each one to simulate diagnostic test data that were then analyzed using BLCA. Each parameter set included the sample size, sensitivity and specificity values for three hypothetical diagnostic tests and the “true” underlying infection prevalence in a hypothetical sample population. Tests 1 and 2 had Se/Sp fixed at 70%/70% and 80%/80%, respectively (red squares in Figure 1b), and these values remained constant for all data simulations. Test 3 was selected sequentially from points A‐E, such that the fixed specificity provided to the model decreased from 100% to 80% (A‐E in Figure 1b). We simulated datasets using these five initial diagnostic test selections ((test1, test2) x (test A‐E)), seven sample sizes (*n* = 20, 40, 80, 160, 320, 640 and 1,280) and three prevalence levels spanning a broad range of ecological scenarios (10%, 50%, 90%), resulting in 105 unique parameter sets. To assess whether observed patterns were influenced by the initial choices for tests 1 and 2 (which had Se:Sp ratios of 1:1), the following two alternate selections for these tests were used: Se/Sp for tests 1 and 2 set to 90%/70% and 70%/90%, respectively (Figure S1b), and the Se/Sp for tests 1 and 2 set to 80%/60% and 50%/90%, respectively (Figure S1c). These alternative scenarios explore different Se:Sp ratios as well as different overall quality of tests 1 and 2.

### 2.2 Data simulation

**FIGURE 2:**
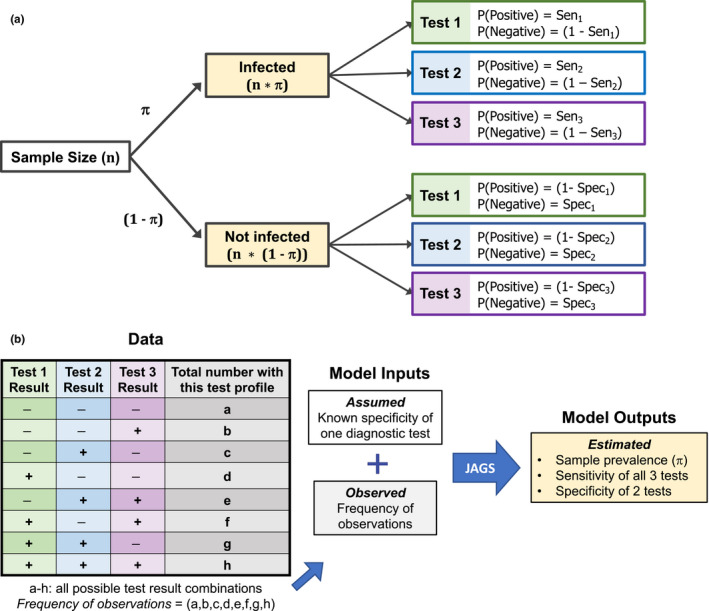
Possible infection categories and test results for a sample population (a), and the workflow for assessment in Bayesian latent class analysis (b). (a) The relationship between sample size, prevalence, and the probability of a positive or negative result for three different diagnostic tests. For infected individuals, the probability of a given test result is proportional to the sensitivity (Se) of that test (top right). For uninfected individuals, the probability of a given test result is proportional to the specificity (Sp) of that test (bottom right). (b) Workflow diagram for Bayesian latent class analysis, taking results from data along with the fixed specificity of one test to obtain posterior probability estimates for all unknown (latent) parameters

For each parameter set (i.e., sample size, prevalence, and hypothetical test combination), a number of individuals (equal to sample size*prevalence) were assigned the status infected, and all remaining individuals in the population were assigned the status uninfected (Figure 2a). Once infection status was set, a series of Bernoulli trials was used to simulate the outcome of each hypothetical diagnostic test. Among infected individuals, the probability of a positive result was equal to test sensitivity, and the probability of a negative result was equal to (1‐Se). Among uninfected individuals, the probability of a positive result was equal to (1‐Sp), and the probability of a negative result was equal to test specificity. These simulations generated a set of three binary test outcomes for each individual, assuming independence among tests, with eight possible combinations of positive and negative test results (ranging from all negative to all positive). The number of individuals that fell into each of the eight possible test result combinations was counted (test profiles a‐h; Figure 2b), and this vector was saved to input in the BLCA model. Test results were simulated 1,000 times for each set of parameters.

### 2.3 Bayesian latent class analysis

Bayesian latent class analysis is a likelihood‐based statistical method that estimates the prevalence of particular class types within a population sample. Here, individuals fall into one of eight observed classes (a‐h), based on the profile of their diagnostic test outcomes (Limmathurotsakul et al., 2012; Figure 2b). Our model assumes the outcome of each diagnostic test is independent of the others, conditional on the individual's underlying (and unknown) state with respect to pathogen infection and disease. Thus, the probability of obtaining a given diagnostic profile depends on the probability that an individual was truly infected (equal to population prevalence) and on the outcome of each diagnostic test given the underlying infection status. As sensitivity is defined as the probability of detecting true positives and specificity is defined as the probability of detecting true negatives, the probability of three negative test outcomes (diagnostic profile a), is:

$$p\left(a\right)=\pi \left(1-{\text{Se}}_{1}\right)\left(1-{\text{Se}}_{2}\right)\left(1-{\text{Se}}_{3}\right)+\left(1-\pi \right)\left({\text{Sp}}_{1}\right)\left({\text{Sp}}_{2}\right)\left({\text{Sp}}_{3}\right).$$

where *π* denotes prevalence, Se_1_ denotes the sensitivity of test 1, Sp_1_ denotes the specificity of test 1, and so on. The first term in this expression represents the probability of being infected and having a false‐negative result for all three tests, while the second term represents the probability of being uninfected and having a true‐negative result for all three tests. Similar logic can be used to find the probability of each diagnostic profile (b‐h, Figure S2), and the observed distribution of diagnostic profiles can be modeled by a multinomial likelihood, with probabilities for each class given by {*p*(*a*),*p*(*b*),…*p*(*h*)} (Rindskopf & Rindskopf, 1986).

### 2.4 Parameter estimation

**FIGURE 3:**
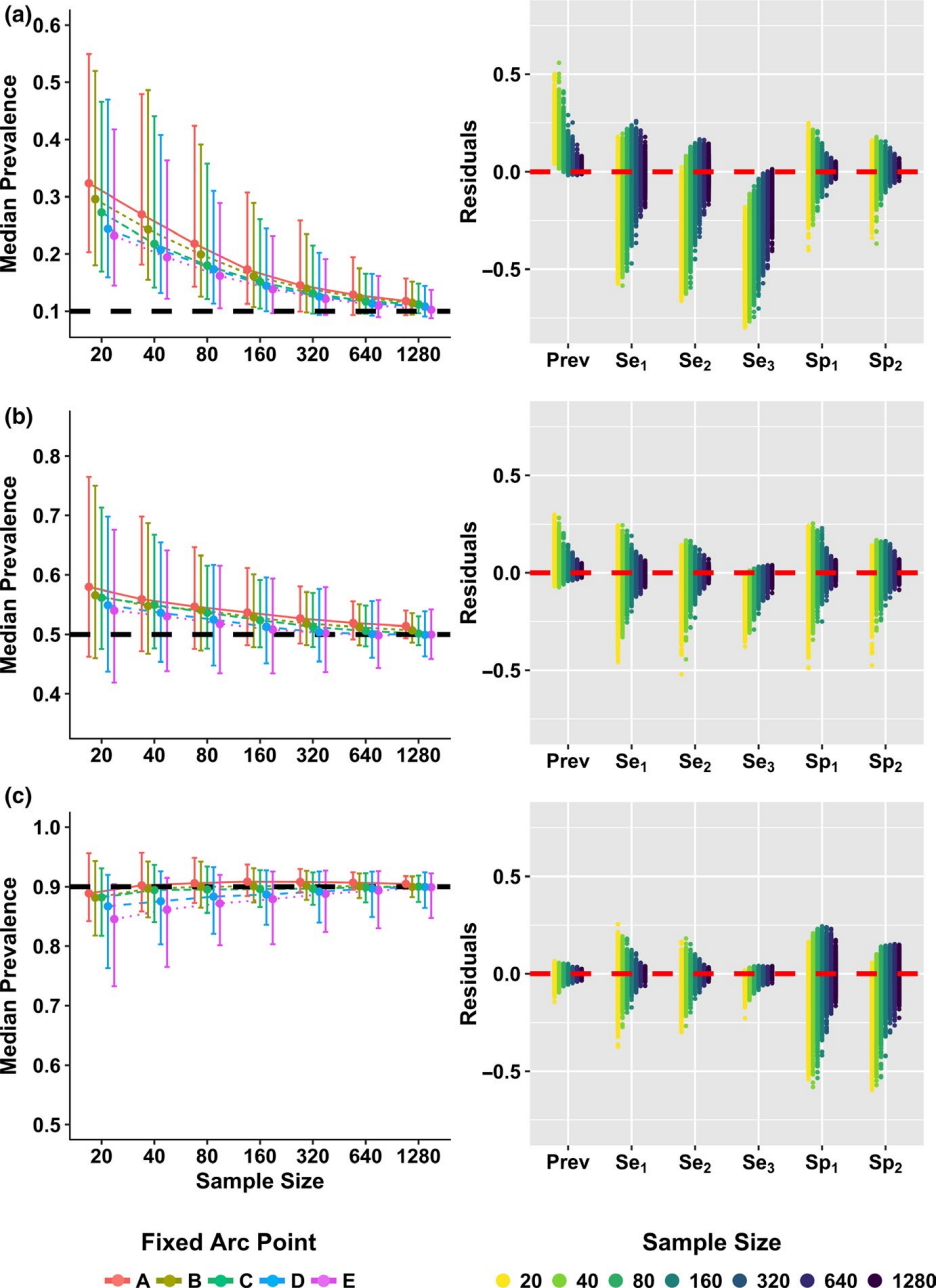
Parameter estimates at three true prevalence levels (10%, 50%, and 90%). *Left:* Median prevalence estimates and 95% credible intervals (CrI) are shown for points A‐E at a true prevalence of 10% (a), 50% (b), and 90% (c), with true prevalence shown as dashed black lines (*y*‐axes scaled equally). *Right:* Residuals for all parameter estimates (prevalence, sensitivities for tests 1–3, specificities for tests 1 and 2) using simulated samples (*n* = 1,000) generated with fixed arc point C (test 3 fixed Se = 95% and Sp = 95%), with zero shown as dashed red line

We estimated parameters in a Bayesian framework using Markov chain Monte Carlo (MCMC). We ran three chains for 10,000 iterations each, with the first 5,000 steps discarded as burn‐in. Uninformative priors (uniform distributions on [0,1]) were assumed for the prevalence, sensitivity of tests 1–3, and specificity of tests 1 and 2 (Figure S2). The fixed specificity for test 3 (one value from points A‐E; Figure 1b) and the frequency of each test profile type (frequency of observations) were used as model inputs (Figure 2b). We modified Bayesian inference code (WinBUGS (Lunn, Thomas, Best, & Spiegelhalter, 2000)) from a previous study (Limmathurotsakul et al., 2012), and JAGS (Plummer, 2003) model estimation was performed using the package R2jags (R2jags, Su, & Yajima, 2015) in R (R Foundation for Statistical Computing, 2016; version 3.3.2). We checked that the Gelman and Rubin statistic was < 1.1 to verify convergence of all MCMC chains (Gelman, Carlin, Stern, & Rubin, 2003) and reported the median and marginal composite 95% credible interval (CrI) for all estimated parameters. Prevalence estimates and 95% CrI were computed for all hypothetical test sets (left panels of Figure 3, S3 and S4), and residuals for all estimated parameters were computed for the fixed test assuming the sensitivity and specificity combination at arc point C (Se_3_ = 0.95/Sp_3_ = 0.95; right panels of Figure 3, S3 and S4). We ran additional simulations using informed priors to determine how an investigator's prior knowledge or suspicion of low, medium or high prevalence levels in a system would affect the estimates of prevalence obtained from BLCA (π ~ beta(2,9), beta(9,9) and beta(9,2) for low, medium, and high prevalence, respectively; Figure S5). Results were compared to the original estimates obtained using uninformative priors (Figure S6).

### 2.5 Wildlife case study

To assess BLCA in a wildlife dataset, we analyzed results of three different tests used to determine *Leptospira* infection status in California sea lions admitted to The Marine Mammal Center (TMMC). TMMC is a marine mammal rehabilitation center that maintains a detailed database of health and medical diagnostic records for individual marine mammals stranding along the California coast. Clinical *Leptospira* infections are diagnosed by clinicians at TMMC using the following diagnostic criteria: high serum MAT antibody titers (>1:3,200) against serovar Pomona, *Leptospira* DNA present in urine or kidney samples (tested via PCR; Wu et al., 2014), or serum chemistry markers indicative of kidney dysfunction (BUN > 100 mg/dl, creatinine > 2 mg/dl, sodium > 155 meq/L and phosphorus > calcium; Colagross‐Schouten, Mazet, Gulland, Miller, & Hietala, 2002; Greig et al., 2005). In this system, we judged that conditional independence among tests was a reasonable assumption, due to the different biological systems targeted by these three diagnostic tests (humoral immune response, presence of pathogen DNA in the urinary tract, and measures of renal function, respectively). To minimize the effects of clinical treatment on test outcomes, we selected only California sea lions at TMMC that had test results for all three *Leptospira* diagnostics from samples collected within one week of admission (*n* = 290; years: 2006–2016). We summed the total number of animals with each test result profile (the frequency of observations) and fixed the specificity of test 3 (PCR) to 97.2% based on a recent estimate for *Leptospira* in humans (Limmathurotsakul et al., 2012). While the PCR method utilized here was previously reported with 100% analytic specificity in CSL urine or kidney tissue (Wu et al., 2014), we chose this slightly more conservative specificity level to reflect the possibility that sample contamination could lead to rare false positives. Parameter estimation was conducted as described above using R2jags, yielding median estimates with 95% CrIs for all unknown parameters.

To test model performance under these estimated real‐world conditions, we simulated CSL data (*n* = 300) using our best parameter estimates as known parameter values (“Values Selected for CSL Simulated Data” in Table 1), then used BLCA on the simulated CSL data to see how accurate model estimates were across 1,000 simulations (Table 1). To assess when BLCA prevalence estimates would be preferable to those obtained using the single best diagnostic test, we compared BLCA estimates from our initial hypothetical test set (Figure 1b) to results generated solely from the single best test (points A, C, and E), which were simulated by Bernoulli trials as described above (Tables 2 and S2).

**TABLE 1:** BLCA median parameter estimates and 95% CrIs obtained from three *Leptospira* diagnostic test results in California sea lions (left)

	BLCA Estimates from California sea lion data	Values selected for CSL simulated data	CSL simulated data
Prevalence (*π*)	20.2% (15.6−25.5%)	20%	20.6% (15.8−26.2%)
Sensitivity – MAT (Se_1_)	64.4% (52.0−78.1%)	65%	64.0% (50.5−76.6%)
Sensitivity – SC (Se_2_)	61.1% (48.2−74.3%)	61%	60.0% (46.7−72.7%)
Sensitivity – PCR (Se_3_)	96.0% (86.4−99.9%)	96%	93.9% (90.3−99.6%)
Specificity – MAT (Sp_1_)	98.1% (95.8−99.6%)	98%	98.0% (95.3−99.7%)
Specificity – SC (Sp_2_)	93.2% (89.6−96.3%)	93%	92.9% (89.0−99.7%)
Specificity – PCR (Sp_3_)	NA (fixed at 97.2%)	Fixed at 97.2%	NA (fixed at 97.2%)

Note

These estimated values were chosen as set values for a CSL data simulation (middle). BLCA parameter estimates were then calculated from this simulated CSL data to see how well the model performed (right).

**TABLE 2:**
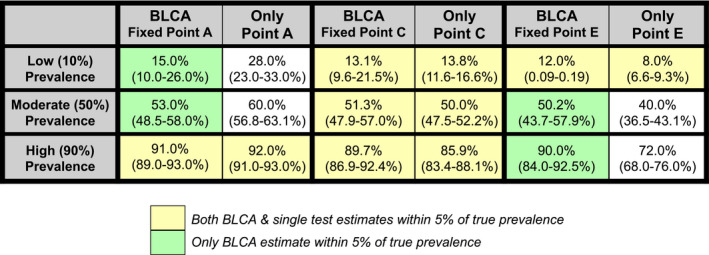
Comparison of prevalence estimates from BLCA versus a single test (sample size, *n* = 320)

Note

Both BLCA and single test estimates within 5% of true prevalence. Only BLCA estimate within 5% of true prevalence. The BLCA estimates were obtained using the original test 1 (Se_1_ = 70%/Sp_1_ = 70%) and test 2 (Se_2_ = 80%/Sp_2_ = 80%) settings, along with point A (left; Se_3_ = 100%/Sp_3_ = 80%), point C (middle; Se_3_ = 95%/Sp_3_ = 95%), or point E (right; Se_3_ = 80%/Sp_3_ = 100%). Single test estimates and 95% CI were obtained using 1,000 Bernoulli trials weighted by the test Se/Sp for test A, C, or E alone. Scenarios where both BLCA and single test estimates were within 5% of the true value are shown in yellow, while scenarios where BLCA alone was within 5% of the true prevalence are shown in green.

## 3 RESULTS

### 3.1 Simulation study

For all simulated scenarios (i.e., all prevalence levels and all hypothetical 3‐test combinations), BLCA prevalence estimates converged on the correct value as the sample size grew (Figure 3). There was some directional bias in prevalence estimates, particularly at low sample sizes, that varied depending on the true infection prevalence. Prevalence of infection was consistently overestimated when infections were rare (true prevalence = 10%) and to a lesser degree when infections were moderately common (true prevalence = 50%). At these prevalence levels, when we varied the specificity of fixed test 3 according to arc points A‐E (Figure 1b), tests with higher specificity returned more accurate estimates at lower sample sizes, although credible intervals across these tests largely overlapped (Figure 3, S3 and S4; Table S1). These patterns were reversed when infections were common (true prevalence = 90%), with prevalence being slightly underestimated and higher sensitivity tests returning more accurate estimates at lower sample sizes, although again credible intervals across these tests largely overlapped (Figure 3, S3 and S4; Table S1).

When infections were rare (true prevalence = 10%), the 95% CrIs for prevalence did not contain the true value until sample size was relatively large (*n* ≥ 160; 95% CrIs for points D & E). At the largest sample sizes (*n* > 320) the true value was contained within the 95% CrIs for all points, and median prevalence estimates were within 3% of the true value (in absolute terms). When true prevalence was moderate (50%), the true value was contained in the 95% CrIs at all sample sizes, and median prevalence estimates were within 8% of the true value at all sample sizes and within 2% at the highest sample sizes (*n* = 640 & *n* = 1,280; Table S1B. In contrast, at higher true prevalence (90%) where prevalence was underestimated at lower sample sizes, the 95% CrIs always contained the true value and prevalence estimates converged quickly to the true prevalence value across all hypothetical test sets (Figure 3c, S3C and S4C; Table S1C).

As with the prevalence estimate, the BLCA estimates of the sensitivity and specificity of each test became more precise and accurate as sample sizes increased (right panels of Figure 3, S2 and S3). However, there were directional biases in these estimates, which exhibited more complex structure than the biases of prevalence estimates. Test sensitivity tended to be underestimated when true prevalence was low, while specificity was underestimated at high prevalences (Figure 3, S2 and S3). When infections were rare (true prevalence = 10%), specificity estimates were more accurate and precise across all sample sizes than sensitivity estimates, while sensitivity estimates were more accurate and precise than specificity estimates when infections were common (true prevalence = 90%; Figure 3, S2 and S3). The residuals of both sensitivity and specificity estimates were generally symmetric, indicating little bias, when infection level was moderate (true prevalence = 50%; Figure 3, S2 and S3).

Considering the potential trade‐off between sensitivity and specificity of a given test (i.e., from tuning the threshold value used to classify a result as positive; Figure 1), we found that the optimal parameters of the best test depend on infection prevalence. When true prevalence is low (10%) or moderate (50%), a fixed specificity of 1.0 of the best test (Point E) yields the most accurate estimate of prevalence (Figure 3a,b). However, when prevalence is high (90%), a fixed sensitivity of 1.0 of the best test (Point A) is preferable (Figure 3c). The influence of this trade‐off is greatest at low prevalence (10%) and weakest at high prevalence (90%) where any Point (A‐E) gives a reasonable prevalence estimate (Table 2).

These broad patterns remained the same regardless of the hypothetical test set used. That is, as the parameters of the two lower‐quality tests change (Figure S1), the patterns of prevalence, sensitivity, and specificity estimation did not vary qualitatively (Figures S3 and S4). However, the quantitative results were noticeably worse (i.e., larger residuals and larger sample sizes needed for accuracy) when these two tests had lower sensitivity and specificity (Figure S4). When we used informative priors in the BLCA to represent investigator knowledge of the prevalence level, estimates of prevalence improved if the prior was close to the true prevalence level, but worsened if the prior was not close to the true prevalence value (Figure S6). Adjusting the prevalence prior did not qualitatively alter the estimates of other parameters.

### 3.2 Wildlife case study

Results from the sea lion case study were concordant with our analyses of the broader simulated data. Although disease prevalence was low in the sea lion system, our sample size was well within the range at which BLCA could produce accurate prevalence estimates using simulated CSL data. The estimated prevalence of clinical *Leptospira* infections in this sample of California sea lions was 20.2% (95% CrI, 15.6%–25.5%; Table 1). Estimates of PCR, MAT and serum chemistry relative test accuracy were broadly consistent with expert knowledge (Table 1). Marine mammal veterinarians consider PCR the best diagnostic test for leptospirosis in sea lions, whereas MAT and serum chemistry are known to be less sensitive and typically used as second‐line tests when urine samples cannot be obtained for PCR.

We also simulated data to verify that BLCA was accurate when using parameters and sample sizes consistent with the best estimates returned by real CSL data. Using these simulated data, the median BLCA prevalence estimate was 20.6% (95% CrI, 15.8%–26.2%; Table 1; Figure S7B), and differed negligibly from the true input prevalence (20%). Sensitivity and specificity values were slightly underestimated, but always within 2.1% of the true value (Table 1). Although this test of simulated data returned very accurate estimates, the direction and magnitude of observed errors were consistent with the error structures reported above for data simulated using other parameters.

### 3.3 Comparing BLCA to results of a single diagnostic test

The BLCA prevalence estimate for the California sea lion sample (20.2%) was very similar to the crude estimate obtained from PCR alone (62/290 positive; 21.4%). This prompted us to consider the marginal value of BLCA and whether it was worth the additional effort. In particular, we explored the circumstances under which the 3‐test BLCA prevalence estimates would improve upon results from a single best test, exploring the influence of the trade‐off between sensitivity and specificity of the best test by considering points A, C, and E from our simulation analysis. At a sample size similar to our CSL case study (*n* = 320), prevalence estimates obtained using BLCA and using the single best test (points A, C, or E alone) were comparable in most cases, but in several scenarios BLCA was clearly superior (Table 2). A single test at point A (Se_3_ = 0.8/Sp_3_ = 1) alone overestimated prevalence at low and mid true prevalence levels, while a single test at point E (Se_3_ = 1/Sp_3_ = 0.8) alone underestimated prevalence at mid and high true prevalence levels; in contrast, BLCA was accurate in both these scenarios (Table 2). Thus, the BLCA method can yield stabilizing estimates that are more robust to fluctuations in prevalence than estimates produced by any single test with unbalanced sensitivity and specificity (Tables 2 and S2). These stabilizing effects of BLCA would be particularly useful in a system with cyclical outbreaks.

When comparing BLCA to single test estimates across all sample sizes, these broad patterns held for larger sample sizes, but differed at lower sample sizes (*n* < 320; Table S2). Regardless of sample size, a balanced high‐quality test with very high sensitivity and specificity (test C) is comparable to BLCA. However, at high sample sizes BLCA converges on the true value at all prevalence levels whereas test C alone converges on over‐ or under‐estimates of prevalence. At 50% prevalence, test C converges on the true estimate, but this is due to canceling of symmetric errors from its identical sensitivity and specificity values.

Bayesian latent class analysis also usually outperformed estimates from a single test when test specificity or sensitivity was low (closer to points A or E; Figure 1b), but neither method worked well when prevalence, test specificity, and sample size were all low. Thus, in circumstances when sample size and disease prevalence are both low, we recommend choosing a diagnostic test threshold that optimizes test specificity, as this can improve the performance of both BLCA and of the single‐test method (Table S2).

## 4 DISCUSSION

Estimating infection prevalence is challenging in wildlife disease systems, where researchers are often confronted with limited sample sizes and imperfect diagnostic tests that lack species‐specific validation. Here, we have explored the utility of Bayesian latent class analysis (BLCA) as a technique to improve estimates of prevalence and of diagnostic test sensitivity and specificity. We have assumed conditional independence among test results, which is reasonable for the biological system we examined due to differences in the biological systems targeted by our diagnostic assays and our lack of longitudinal sampling (Kostoulas et al., 2017; Wang & Hanson, 2019), but in situations where this is not the case the conditional dependence structure should be considered (Albert & Dodd, 2004; Dendukuri & Joseph, 2001; Hadgu & Qu, 1998; Jones et al., 2010; Pepe & Janes, 2006; Qu et al., 1996). Using simulated data and a case study to explore the utility of BLCA, we demonstrate that the accuracy of prevalence estimates depends on multiple factors: the sample size being tested, the true prevalence in the study system and the sensitivity/specificity of the diagnostic tests being used. We compare BLCA prevalence estimates to those from a single test, demonstrating the stabilizing effects of the BLCA method under different sample sizes and prevalences. In addition, recognizing that many diagnostic tests have an intrinsic trade‐off between sensitivity and specificity (which can be tuned by altering the threshold value used to define a positive test result), we show how the accuracy of prevalence estimates can be optimized depending on the epidemiological context.

The precision and accuracy of parameter estimates increased with sample size across all simulations, providing accurate estimates at large sample sizes regardless of the true infection prevalence (Figure 1, S2, S3, S7). The use of informed priors has the potential to further improve prevalence estimates, highlighting the potential for this Bayesian framework to incorporate expert knowledge from the field. However, in the absence of accurate prior information the use of uninformed priors provides more stable prevalence estimates (Figure S6). We observed directional biases in the prevalence, sensitivity, and specificity estimates depending on whether infections are common (high prevalence) or rare (low prevalence; Greiner & Gardner, 2000). For example, when sample size is relatively low, an overrepresentation of false positives can elevate prevalence estimates when diseases are rare. Conversely, an overrepresentation of false negatives can bias prevalence estimates downward when diseases are common.

Our work demonstrates the potential to improve the accuracy of prevalence estimates by altering the threshold for positivity for the highest quality test (Figure 1a). If results from the best test (the test with the fixed specificity provided to the BLCA model) are quantitative, choosing a threshold that maximizes specificity will improve prevalence estimate accuracy when infections are rare, while maximizing sensitivity will improve estimates when infections are common (Figure 3, S2 and S3; Table S2). This choice follows naturally, because higher specificity allows you to detect more true negatives, which are prevalent when infections are rare. In contrast, higher sensitivity allows you to detect more true positives, which are prevalent when infections are common. In addition, this logic can guide the choice of single tests (or the choice of a threshold for a single test) to use for estimation of prevalence without the need for advanced statistical analysis: our work shows that maximizing test sensitivity for common diseases, or test specificity for rare diseases, can produce single test estimates of comparable accuracy to BLCA.

Analyzing our wildlife case study of *Leptospira interrogans* in California sea lions, we report new estimates for the sensitivity and specificity of key diagnostic tests in this system to explore the statistical power of BLCA for a given sample size. The samples used in this study span a ten‐year period across a range of different epidemiologic conditions, so here our prevalence estimates reflect the prevalence in the sample of stranded animals rather than the prevalence in the wild population at any point in time. At a titer cutoff 1:3,200, our estimates for the sensitivity and specificity of MAT were 64.4% (95% CrI: 52%‐78.1%) and 98.1% (95% CrI: 95.8%‐99.6%), respectively, which differ from previous CSL estimates using this titer cutoff obtained from known positive and negative animals (Se = 100% and Sp = 100%; Colagross‐Schouten et al., 2002). These previous estimates were likely idealized due to small samples and the study design (Greiner & Gardner, 2000), as the negative controls were born in captivity with no possibility of residual titers from a previous exposure, and the positive animals were selected based on clear clinical signs and renal lesions indicating leptospirosis. Diagnosis in wild animals is likely to be complicated by residual titers from previous exposures, or by chronic infections that are no longer associated with a high titer (Buhnerkempe et al., 2017). Due to these and other complicating factors, sensitivity and specificity are unlikely to be perfect in stranded wild animals.

This contrast highlights the influence of the underlying study population and the importance of considering system‐specific characteristics and ecological context when utilizing BLCA. Test sensitivity and specificity estimates likely vary with underlying prevalence and sample size due to the probability of sampling individuals that are truly infected or truly uninfected, which in turn modulates the ratio of false positives to false negatives in the data. For example, at 90% true prevalence, most individuals will be true positives, so testing regimes will have the potential to pick up more true positives/false negatives and fewer true negatives/false positives, leading to a higher estimated sensitivity and lower estimated specificity.

Broadly, we demonstrate that BLCA works well for estimating prevalence and test accuracy, but some caution is warranted because its performance does not always beat that of the single best available test. In particular, there are scenarios with low sample size and low‐to‐moderate prevalence where a single test with high specificity can yield more accurate prevalence estimates than BLCA. A rule of thumb, apparent in Table S2, is that this can happen when the expected number of infected individuals in the sample is ≤10. When the best test has lower specificity (e.g., Test A in Table S2), neither approach worked well if the expected number of infections is ≤20. In all other situations, prevalence estimates from BLCA are comparable to or better than estimates from a single diagnostic test, and this performance advantage increases as the highest quality diagnostic test decreases in sensitivity or specificity (i.e., moving toward points A or E). Furthermore, prevalence estimates made using BLCA will be more robust to changes in prevalence across cyclical epidemics than estimates made using a single test. Our data simulations provide quantitative insight into the relative performance of these approaches, to help researchers assess whether the additional effort of BLCA is worthwhile. In many circumstances, the BLCA method provides more accurate estimates than researchers would otherwise be able to obtain, making it a worthwhile tool that addresses many challenges faced by disease ecologists.

## CONFLICT OF INTEREST

The authors declare no competing interests.

## AUTHOR CONTRIBUTION

**Sarah K Helman: Conceptualization (lead); Data curation (lead); Formal analysis (lead); Investigation (lead); Methodology (lead); Project administration (lead); Writing‐original draft (lead); Writing‐review & editing (lead). Riley O. Mummah:** Data curation (supporting); Formal analysis (supporting); Investigation (supporting); Methodology (supporting); Project administration (supporting); Writing‐review & editing (supporting). **Katelyn M Gostic:** Data curation (supporting); Formal analysis (supporting); Investigation (supporting); Methodology (supporting); Writing‐review & editing (supporting). **Michael Buhnerkempe:** Conceptualization (equal); Data curation (supporting); Formal analysis (supporting); Investigation (supporting); Methodology (supporting); Writing‐review & editing (supporting). **Katherine C Prager:** Conceptualization (supporting); Data curation (supporting); Formal analysis (supporting); Investigation (supporting); Methodology (supporting); Writing‐review & editing (supporting). **James O Lloyd‐Smith:** Conceptualization (equal); Data curation (supporting); Formal analysis (supporting); Investigation (supporting); Methodology (supporting); Project administration (supporting); Writing‐review & editing (supporting).

## Supporting information

Fig S1

Fig S2

Fig S3

Fig S4

Fig S5

Fig S6

Fig S7

Table S1

Table S2

Supplementary Material

Supplementary Material

Supplementary Material

## Data Availability

All CSL data and the code used to generate additional datasets during this study are included in this published article (and its supplementary information files).
