# Supplementary material for: Estimating prevalence and test accuracy in disease ecology: How Bayesian latent class analysis can boost or bias imperfect test results
Source: Ecol Evol. 2020 Jun 15;10(14):7221–32. doi: 10.1002/ece3.6448 (PMC7391344; doi:10.1002/ece3.6448)
Supplement: Supplementary file 1 — Fig S1 [file ECE3-10-7221-s001.pdf]

A

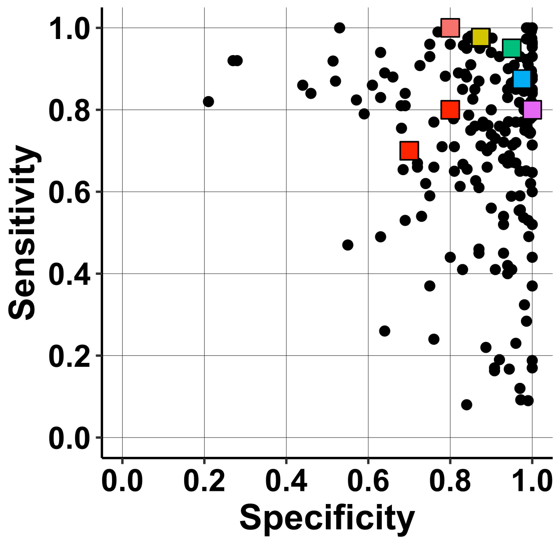

Test 1
- Specificity: 70%
- Sensitivity: 70%

Test 2
- Specificity: 80%
- Sensitivity: 80%

Test 3

Arc Point	Specificity	Sensitivity
A	0.8	1
B	0.875	0.975
C	0.95	0.95
D	0.975	0.875
E	1	0.8

B

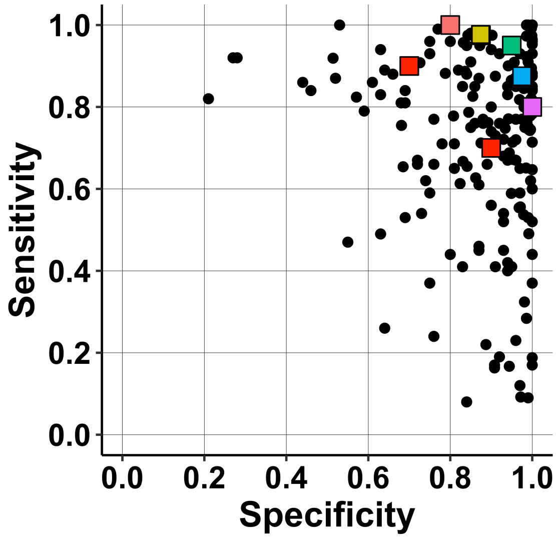

Test 1
- Specificity: 70%
- Sensitivity: 90%

Test 2
- Specificity: 90%
- Sensitivity: 70%

Test 3
- Same as S1A

C

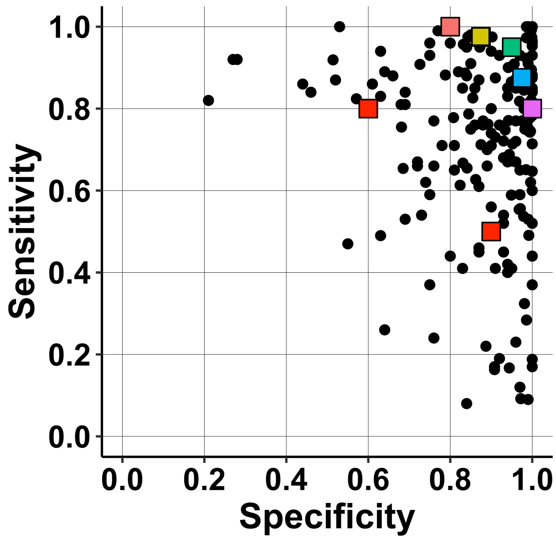

Test 1
- Specificity: 60%
- Sensitivity: 80%

Test 2
- Specificity: 90%
- Sensitivity: 50%

Test 3
- Same as S1A
