# Supplementary material for: Estimating prevalence and test accuracy in disease ecology: How Bayesian latent class analysis can boost or bias imperfect test results
Source: Ecol Evol. 2020 Jun 15;10(14):7221–32. doi: 10.1002/ece3.6448 (PMC7391344; doi:10.1002/ece3.6448)
Supplement: Supplementary file 2 — Fig S2 [file ECE3-10-7221-s002.pdf]

A. JAGS Model

```
model{
#total<-sum(freqobs[1:8])

#Likelihood
freqobs[1:8]~ dmulti(p[1:8],total)

for (i in 1:8){

p[i]<- prev*(positive[i]) + (1-prev)*(negative[i])

positive[i]  <- (Se[1]) * test1[i] + (1-Se[1]) * (1-test1[i])) *
                (Se[2]) * test2[i] + (1-Se[2]) * (1-test2[i])) *
                (Se[3]) * test3[i] + (1-Se[3]) * (1-test3[i]))

negative[i] <- ((1-Sp[1]) * test1[i] + Sp[1] * (1-test1[i])) *
               ((1-Sp[2]) * test2[i] + Sp[2] * (1-test2[i])) *
               ((1-Sp[3]) * test3[i] + Sp[3] * (1 -test3[i]))}

#Priors
prev~dbeta(1,1)
Se[3]~dbeta(1,1)
Sp[3]<- XX

for (j in 1:2){
s[j]~dbeta(1,1)
x[j]~dbeta(1,1) }

}
```

B. JAGS Model Explanation

freqobs: frequency of observations for each test profile (1:8 refers to profiles a-h)

Sum(freqobs[1:8]): total individuals tested

Likelihood

p[i]: probability of each test profile (∑ p[i]=1)

positive[i]: probability that an individual has test profile [i] given the individual is truly positive

negative[i]: probability that an individual has test profile [i] given the individual is truly negative

Tests: JAGS model input designating test results (0=negative, 1=positive). These test vectors jointly represent all possible test profiles.

test1 <- c(0,0,1,1,0,0,1,1)
test2 <- c(0,1,0,1,0,1,0,1)
test3 <- c(0,0,0,0,1,1,1,1)

Priors

Prev: Prevalence (noninformative)
Se[3]: Sensitivity of test 3 (noninformative)
Sp[3]: Specificity of test 3 **(fixed value=XX)**

Se[1:2]: Sensitivity of test 1 & 2 (noninformative)
Sp[1:2]: Specificity of test 1 & 2 (noninformative)

C. Probability Table

Test 1 Result	Test 2 Result	Test 3 Result	Total number with this test profile	Expected Total Number for Each Test Profile<br>$n\pi$: total true positives $n(1-\pi)$: total true negatives
-	-	-	a	$n\pi * (1-Se_1)(1-Se_2)(1-Se_3) + n(1-\pi) * (Sp_1Sp_2Sp_3)$
-	+	-	b	$n\pi * (1-Se_1)Se_2(1-Se_3) + n(1-\pi) * Sp_1(1-Sp_2)Sp_3$
+	-	-	c	$n\pi * Se_1(1-Se_2)(1-Se_3) + n(1-\pi) * (1-Sp_1)Sp_2Sp_3$
+	+	-	d	$n\pi * Se_1Se_2(1-Se_3) + n(1-\pi) * (1-Sp_1)(1-Sp_2)Sp_3$
-	-	+	e	$n\pi * (1-Se_1)(1-Se_2)Se_3 + n(1-\pi) * Sp_1Sp_2(1-Sp_3)$
-	+	+	f	$n\pi * (1-Se_1)Se_2Se_3 + n(1-\pi) * Sp_1(1-Sp_2)(1-Sp_3)$
+	-	+	g	$n\pi * Se_1(1-Se_2)Se_3 + n(1-\pi) * (1-Sp_1)Sp_2(1-Sp_3)$
+	+	+	h	$n\pi * Se_1Se_2Se_3 + n(1-\pi) * (1-Sp_1)(1-Sp_2)(1-Sp_3)$
