# Supplementary figures and images for: Estimating prevalence and test accuracy in disease ecology: How Bayesian latent class analysis can boost or bias imperfect test results
Source: Ecol Evol. 2020 Jun 15;10(14):7221–32. doi: 10.1002/ece3.6448 (PMC7391344; doi:10.1002/ece3.6448)

**A**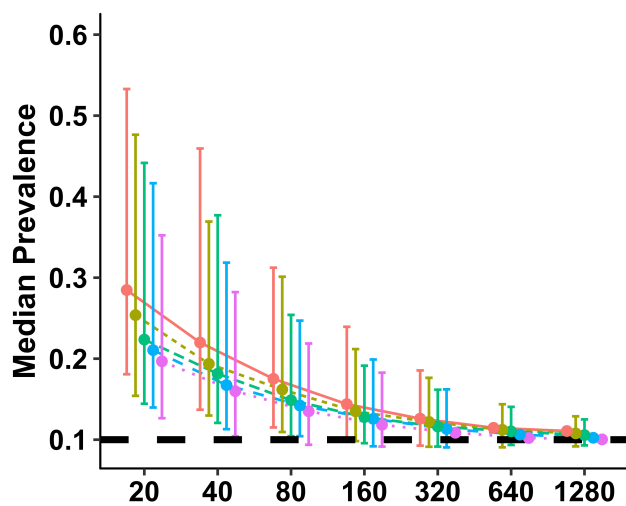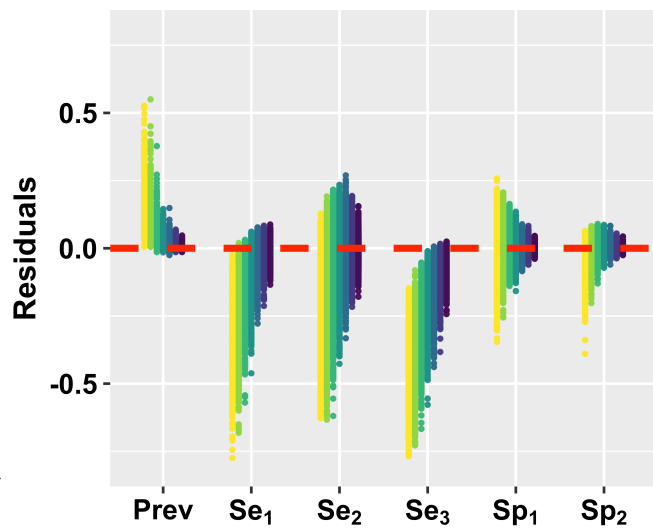**B**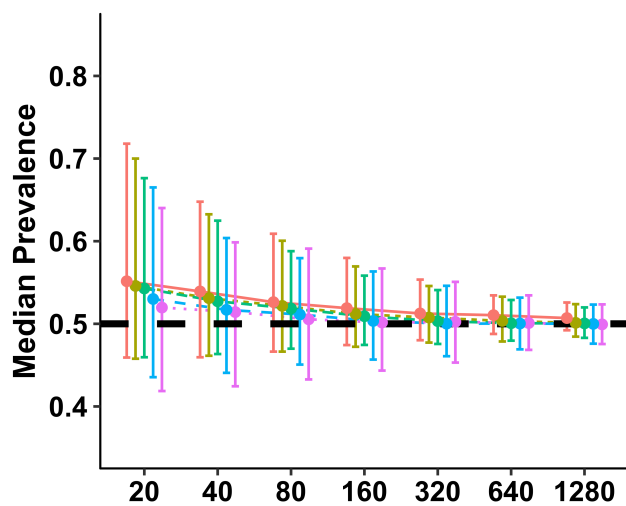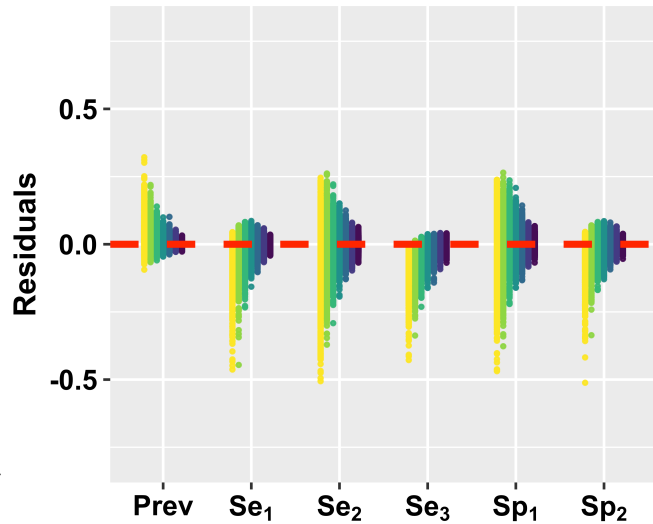**C**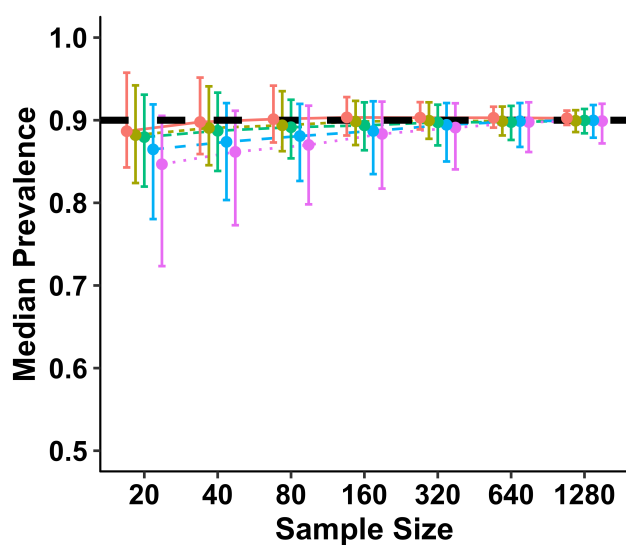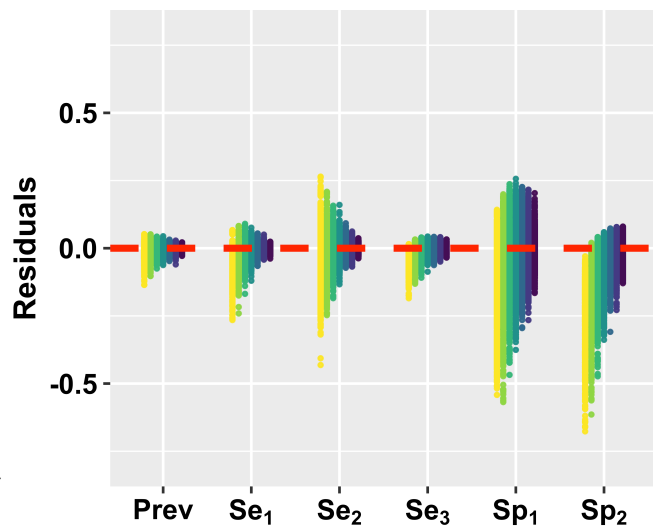**Fixed Arc Point**

A B C D E

**Sample Size**

20 40 80 160 320 640 1280

Supplement: Supplementary file 3 — Fig S3 [file ECE3-10-7221-s003.pdf]

**A**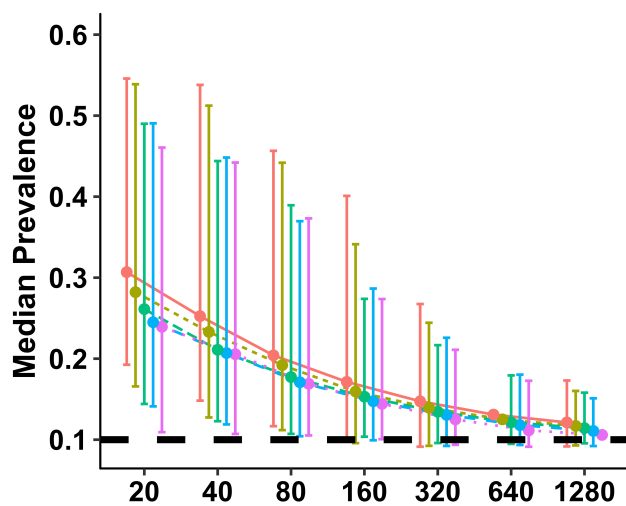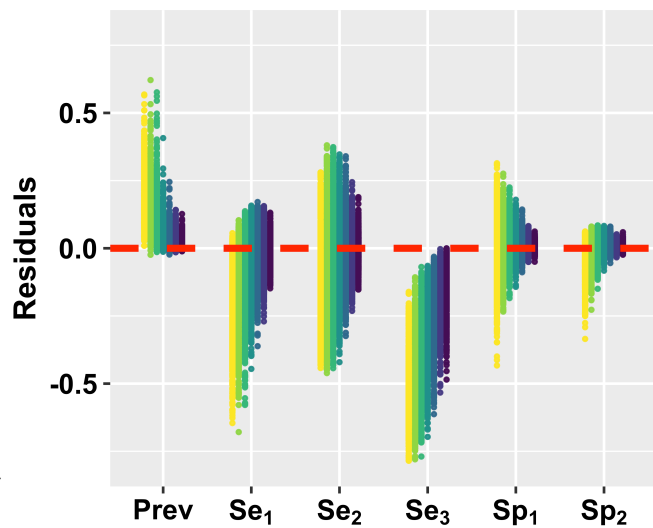**B**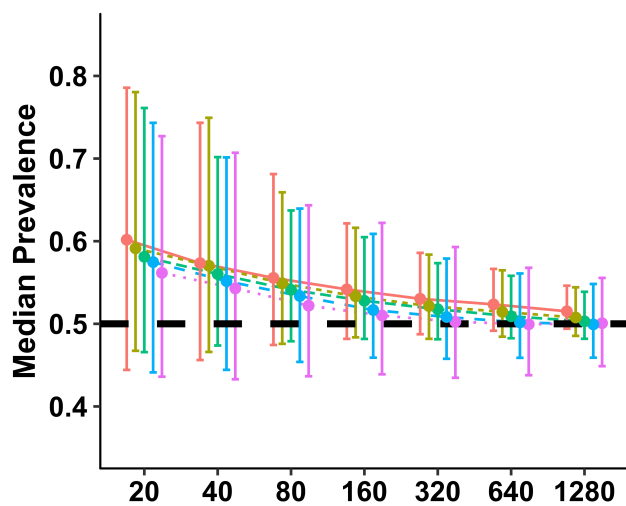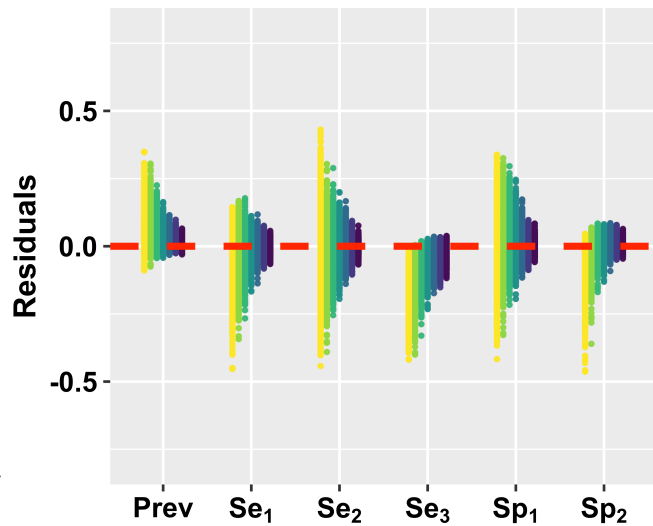**C**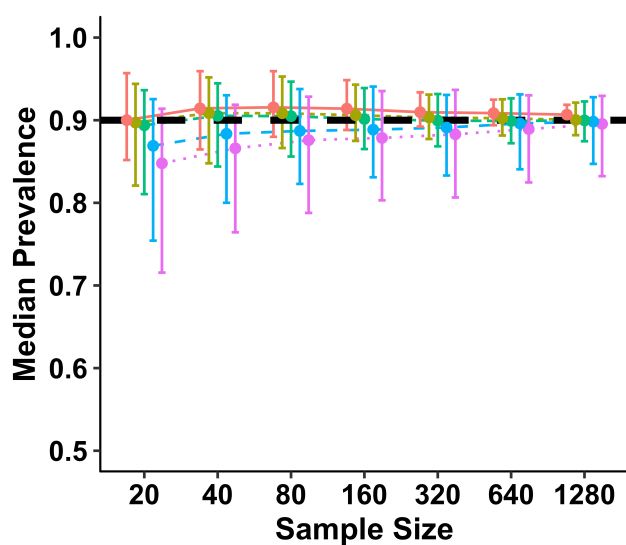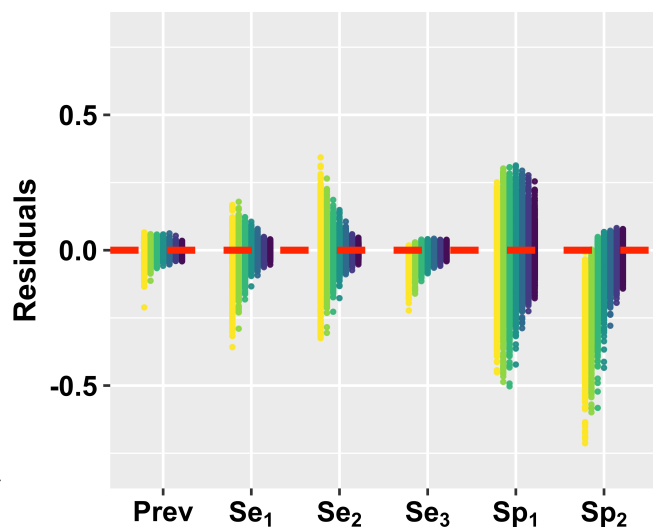**Fixed Arc Point**

A B C D E

**Sample Size**

20 40 80 160 320 640 1280

Supplement: Supplementary file 4 — Fig S4 [file ECE3-10-7221-s004.pdf]

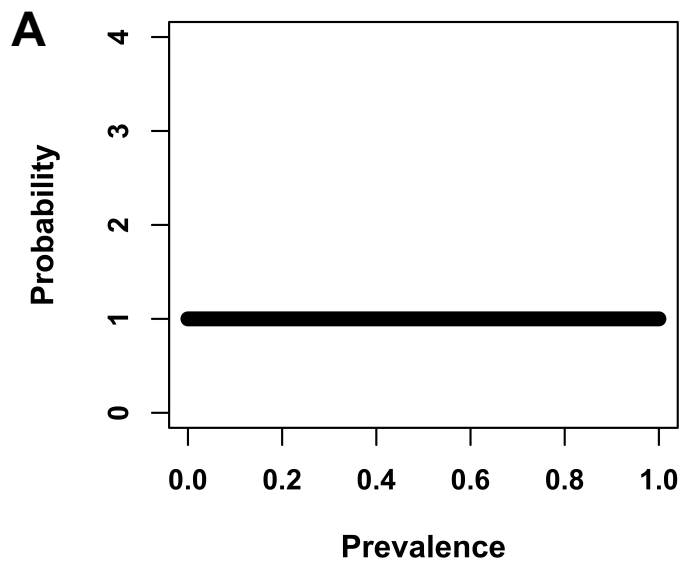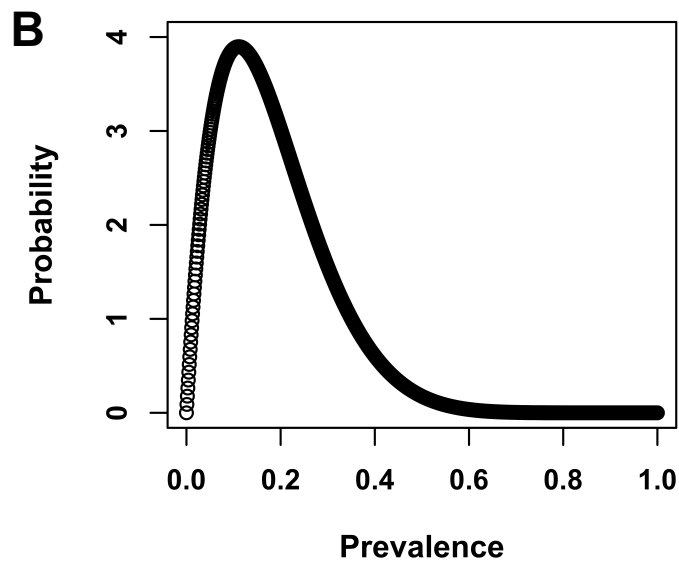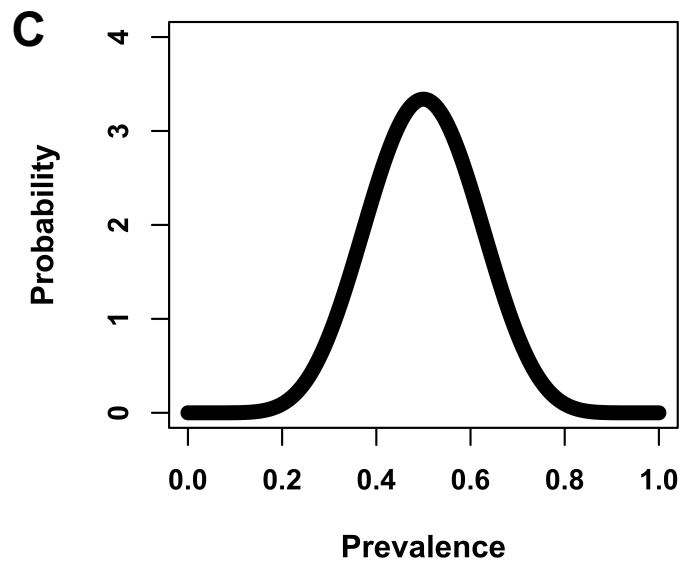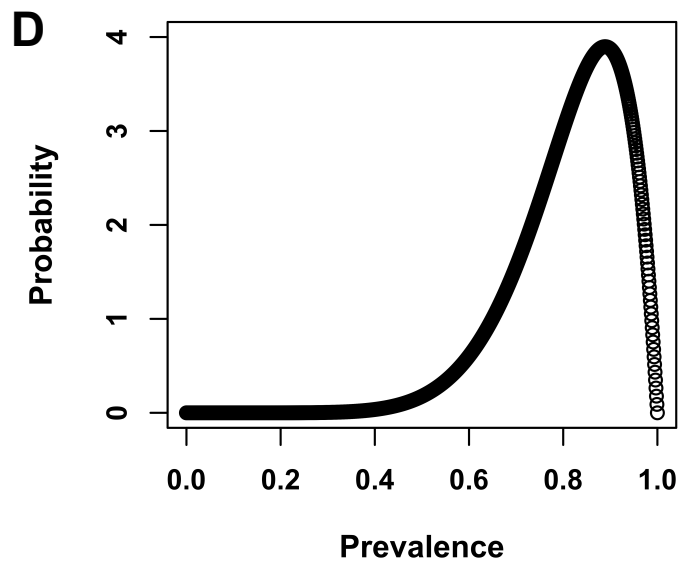

Supplement: Supplementary file 5 — Fig S5 [file ECE3-10-7221-s005.pdf]

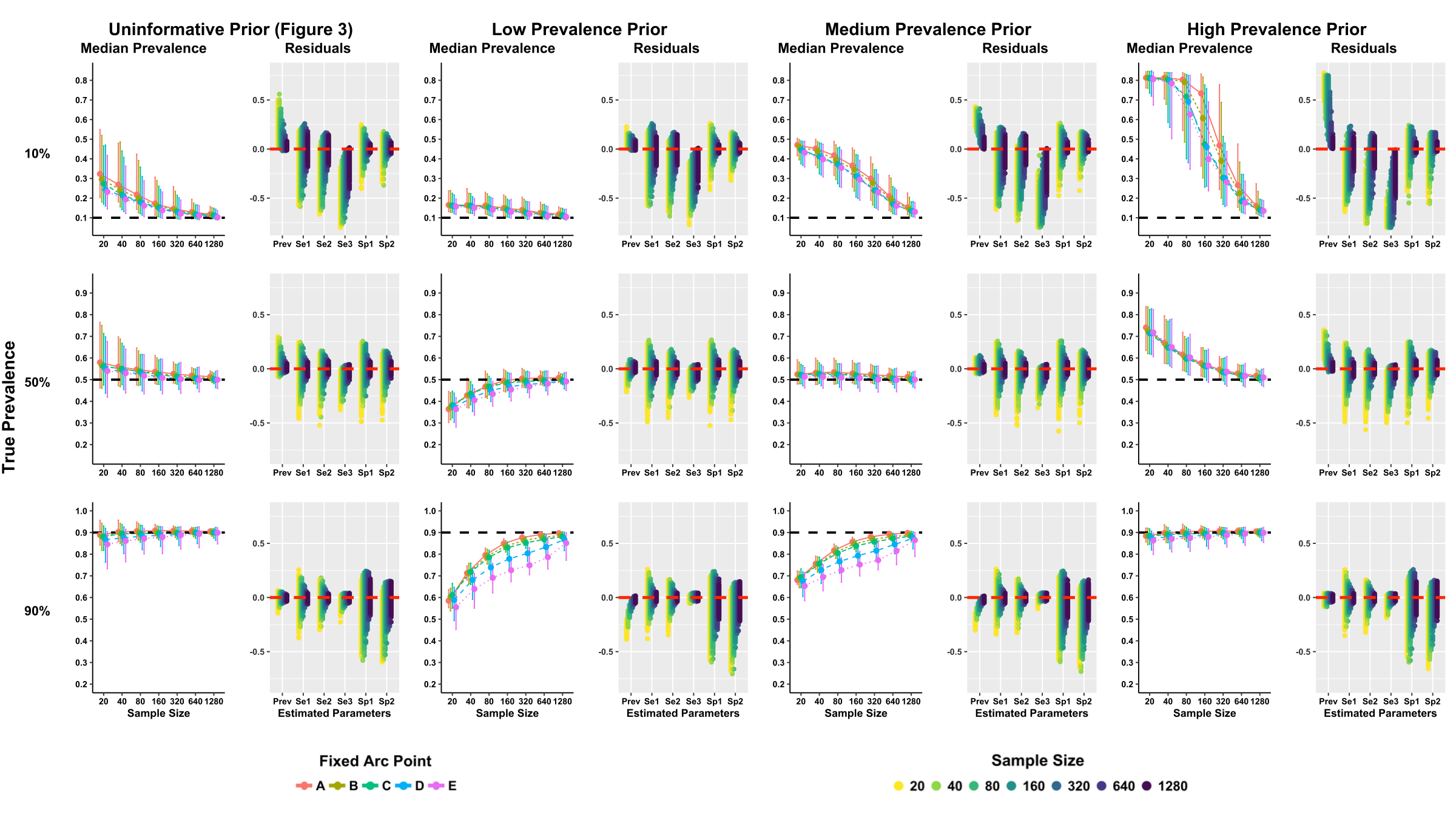

Supplement: Supplementary file 6 — Fig S6 [file ECE3-10-7221-s006.pdf]

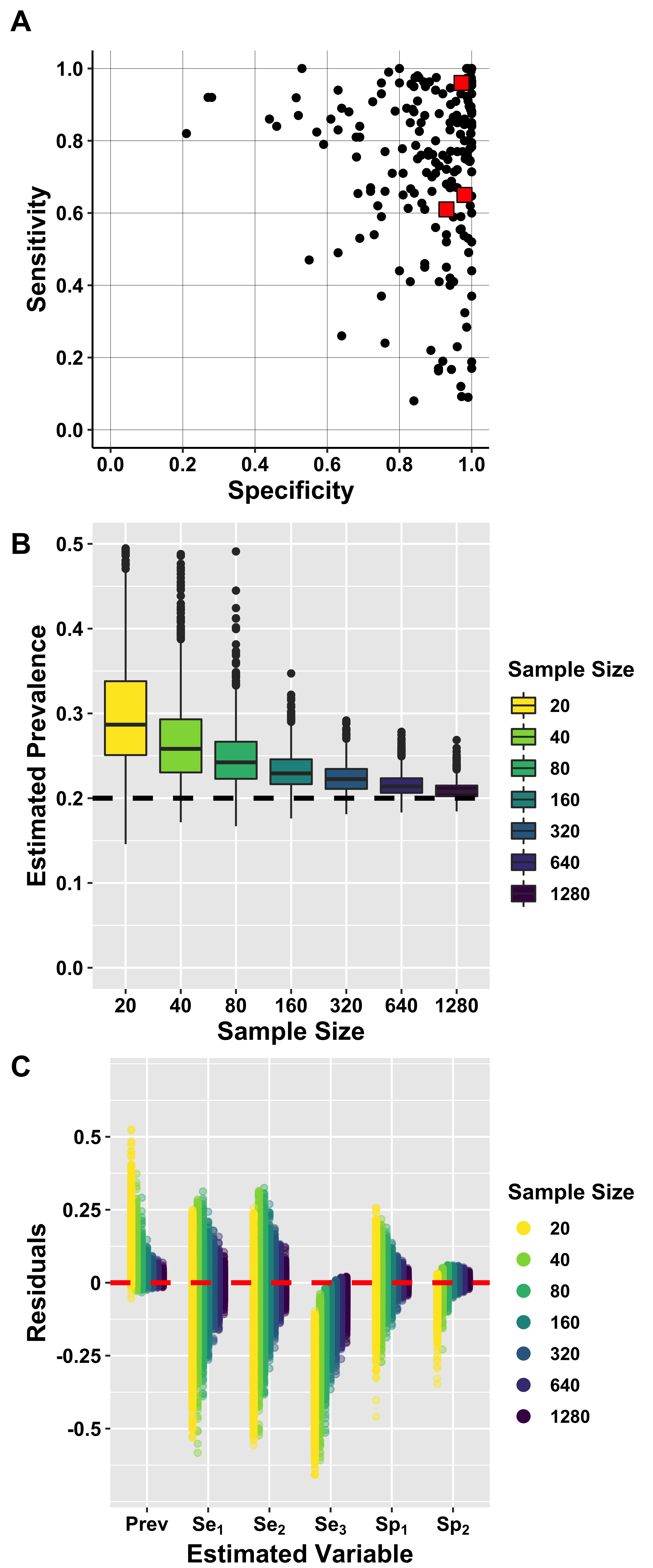

Supplement: Supplementary file 7 — Fig S7 [file ECE3-10-7221-s007.png]
