# Supplementary material for: Estimating prevalence and test accuracy in disease ecology: How Bayesian latent class analysis can boost or bias imperfect test results
Source: Ecol Evol. 2020 Jun 15;10(14):7221–32. doi: 10.1002/ece3.6448 (PMC7391344; doi:10.1002/ece3.6448)
Supplement: Supplementary file 8 — Table S1 [file ECE3-10-7221-s008.pdf]

Table S1A: True prevalence = 10%.

Sample Size	Prevalence *Point A Test 3: Sp=0.8/Se=1*	Prevalence *Point B Test 3: Sp=0.875/Se=0.975*	Prevalence *Point C Test 3: Sp=0.95/Se=0.95*	Prevalence *Point D Test 3: Sp=0.975/Se=0.875*	Prevalence *Point E Test 3: Sp=1/Se=0.8*
**n=20**	0.323 (0.203-0.549)	0.296 (0.18-0.52)	0.273 (0.169-0.466)	0.244 (0.159-0.47)	0.232 (0.145-0.418)
**n=40**	0.269 (0.181-0.479)	0.243 (0.155-0.486)	0.218 (0.141-0.44)	0.207 (0.132-0.408)	0.194 (0.122-0.363)
**n=80**	0.218 (0.143-0.424)	0.199 (0.125-0.391)	0.18 (0.121-0.358)	0.173 (0.113-0.31)	0.162 (0.105-0.289)
**n=160**	0.173 (0.113-0.307)	0.161 (0.107-0.29)	0.151 (0.105-0.261)	0.144 (0.1-0.245)	0.138 (0.097-0.231)
**n=320**	0.145 (0.099-0.259)	0.14 (0.098-0.235)	0.131 (0.096-0.215)	0.125 (0.094-0.202)	0.121 (0.094-0.191)
**n=640**	0.129 (0.093-0.194)	0.124 (0.096-0.175)	0.117 (0.096-0.166)	0.113 (0.092-0.165)	0.11 (0.09-0.162)
**n=1280**	0.118 (0.093-0.157)	0.115 (0.094-0.152)	0.112 (0.097-0.148)	0.108 (0.091-0.144)	0.103 (0.088-0.137)

Table S1B: True prevalence = 50%.

Sample Size	Prevalence *Point A Test 3: Sp=0.8/Se=1*	Prevalence *Point B Test 3: Sp=0.875/Se=0.975*	Prevalence *Point C Test 3: Sp=0.95/Se=0.95*	Prevalence *Point D Test 3: Sp=0.975/Se=0.875*	Prevalence *Point E Test 3: Sp=1/Se=0.8*
**n=20**	0.58 (0.462-0.765)	0.566 (0.46-0.75)	0.562 (0.475-0.713)	0.549 (0.438-0.698)	0.54 (0.419-0.676)
**n=40**	0.559 (0.472-0.698)	0.548 (0.467-0.687)	0.55 (0.476-0.668)	0.536 (0.454-0.655)	0.53 (0.438-0.641)
**n=80**	0.547 (0.475-0.647)	0.541 (0.473-0.633)	0.536 (0.476-0.615)	0.525 (0.448-0.617)	0.518 (0.435-0.615)
**n=160**	0.537 (0.482-0.612)	0.529 (0.478-0.601)	0.524 (0.478-0.591)	0.513 (0.451-0.596)	0.508 (0.434-0.594)
**n=320**	0.527 (0.485-0.58)	0.518 (0.482-0.571)	0.513 (0.479-0.57)	0.504 (0.454-0.576)	0.502 (0.437-0.579)
**n=640**	0.519 (0.491-0.556)	0.512 (0.482-0.551)	0.506 (0.48-0.548)	0.501 (0.46-0.556)	0.498 (0.444-0.558)
**n=1280**	0.514 (0.493-0.54)	0.507 (0.486-0.536)	0.502 (0.482-0.53)	0.499 (0.463-0.539)	0.50 (0.458-0.542)

Table S1C: True prevalence = 90%.

Sample Size	Prevalence *Point A Test 3: Sp=0.8/Se=1*	Prevalence *Point B Test 3: Sp=0.875/Se=0.975*	Prevalence *Point C Test 3: Sp=0.95/Se=0.95*	Prevalence *Point D Test 3: Sp=0.975/Se=0.875*	Prevalence *Point E Test 3: Sp=1/Se=0.8*
**n=20**	0.889 (0.842-0.956)	0.882 (0.818-0.943)	0.882 (0.817-0.931)	0.867 (0.763-0.92)	0.846 (0.733-0.904)
**n=40**	0.902 (0.858-0.957)	0.897 (0.849-0.942)	0.894 (0.84-0.937)	0.875 (0.803-0.926)	0.861 (0.765-0.915)
**n=80**	0.906 (0.873-0.948)	0.9 (0.864-0.942)	0.895 (0.856-0.934)	0.883 (0.821-0.933)	0.872 (0.801-0.92)
**n=160**	0.909 (0.884-0.937)	0.901 (0.873-0.931)	0.896 (0.866-0.928)	0.887 (0.836-0.927)	0.879 (0.803-0.925)
**n=320**	0.908 (0.891-0.93)	0.902 (0.878-0.926)	0.897 (0.869-0.924)	0.892 (0.84-0.925)	0.888 (0.824-0.927)
**n=640**	0.907 (0.895-0.924)	0.901 (0.881-0.922)	0.899 (0.873-0.923)	0.897 (0.849-0.925)	0.894 (0.83-0.926)
**n=1280**	0.905 (0.896-0.918)	0.9 (0.882-0.917)	0.9 (0.874-0.918)	0.9 (0.864-0.924)	0.899 (0.847-0.922)
