# Supplementary material for: Estimating prevalence and test accuracy in disease ecology: How Bayesian latent class analysis can boost or bias imperfect test results
Source: Ecol Evol. 2020 Jun 15;10(14):7221–32. doi: 10.1002/ece3.6448 (PMC7391344; doi:10.1002/ece3.6448)
Supplement: Supplementary file 9 — Table S2 [file ECE3-10-7221-s009.pdf]

**A**

| Sample Size | Prevalence | BLCA - Fixed A | Test A Only | BLCA - Fixed C | Test C Only | BLCA - Fixed E | Test E Only |
|---|---|---|---|---|---|---|---|
| 20 | 0.1 | 0.32 (0.2-0.55) | 0.25 (0.15-0.45) | 0.27 (0.17-0.47) | 0.15 (0.05-0.25) | 0.23 (0.14-0.42) | 0.1 (0-0.1) |
| 40 | 0.1 | 0.27 (0.18-0.48) | 0.28 (0.15-0.4) | 0.22 (0.14-0.44) | 0.12 (0.08-0.22) | 0.19 (0.12-0.36) | 0.08 (0.05-0.1) |
| 80 | 0.1 | 0.22 (0.14-0.42) | 0.28 (0.2-0.36) | 0.18 (0.12-0.36) | 0.14 (0.1-0.19) | 0.16 (0.11-0.29) | 0.09 (0.05-0.1) |
| 160 | 0.1 | 0.17 (0.11-0.31) | 0.28 (0.22-0.34) | 0.15 (0.1-0.26) | 0.14 (0.11-0.18) | 0.14 (0.1-0.23) | 0.08 (0.06-0.09) |
| 320 | 0.1 | 0.15 (0.1-0.26) | 0.28 (0.24-0.32) | 0.13 (0.1-0.21) | 0.14 (0.12-0.16) | 0.12 (0.09-0.19) | 0.08 (0.07-0.09) |
| 640 | 0.1 | 0.13 (0.09-0.19) | 0.28 (0.25-0.31) | 0.12 (0.1-0.17) | 0.14 (0.12-0.16) | 0.11 (0.09-0.16) | 0.08 (0.07-0.09) |
| 1280 | 0.1 | 0.12 (0.09-0.16) | 0.28 (0.26-0.3) | 0.11 (0.1-0.15) | 0.14 (0.13-0.15) | 0.1 (0.09-0.14) | 0.08 (0.07-0.09) |

**B**

| Sample Size | Prevalence | BLCA - Fixed A | Test A Only | BLCA - Fixed C | Test C Only | BLCA - Fixed E | Test E Only |
|---|---|---|---|---|---|---|---|
| 20 | 0.5 | 0.58 (0.46-0.76) | 0.6 (0.5-0.75) | 0.56 (0.48-0.71) | 0.5 (0.4-0.6) | 0.54 (0.42-0.68) | 0.4 (0.25-0.5) |
| 40 | 0.5 | 0.56 (0.47-0.7) | 0.6 (0.52-0.7) | 0.55 (0.48-0.67) | 0.5 (0.42-0.57) | 0.53 (0.44-0.64) | 0.4 (0.32-0.48) |
| 80 | 0.5 | 0.55 (0.48-0.65) | 0.6 (0.55-0.66) | 0.54 (0.48-0.62) | 0.5 (0.45-0.55) | 0.52 (0.43-0.62) | 0.4 (0.32-0.46) |
| 160 | 0.5 | 0.54 (0.48-0.61) | 0.6 (0.56-0.64) | 0.52 (0.48-0.59) | 0.5 (0.47-0.53) | 0.51 (0.43-0.59) | 0.4 (0.36-0.44) |
| 320 | 0.5 | 0.53 (0.48-0.58) | 0.6 (0.57-0.63) | 0.51 (0.48-0.57) | 0.5 (0.48-0.52) | 0.5 (0.44-0.58) | 0.4 (0.37-0.43) |
| 640 | 0.5 | 0.52 (0.49-0.56) | 0.6 (0.58-0.62) | 0.51 (0.48-0.55) | 0.5 (0.48-0.52) | 0.5 (0.44-0.56) | 0.4 (0.38-0.42) |
| 1280 | 0.5 | 0.51 (0.49-0.54) | 0.6 (0.58-0.61) | 0.5 (0.48-0.53) | 0.5 (0.49-0.51) | 0.5 (0.46-0.54) | 0.4 (0.38-0.42) |

**C**

| Sample Size | Prevalence | BLCA - Fixed A | Test A Only | BLCA - Fixed C | Test C Only | BLCA - Fixed E | Test E Only |
|---|---|---|---|---|---|---|---|
| 20 | 0.9 | 0.89 (0.84-0.96) | 0.9 (0.9-1) | 0.88 (0.82-0.93) | 0.85 (0.75-0.95) | 0.85 (0.73-0.9) | 0.75 (0.55-0.85) |
| 40 | 0.9 | 0.9 (0.86-0.96) | 0.92 (0.9-0.95) | 0.89 (0.84-0.94) | 0.88 (0.8-0.92) | 0.86 (0.77-0.91) | 0.72 (0.6-0.82) |
| 80 | 0.9 | 0.91 (0.87-0.95) | 0.91 (0.9-0.95) | 0.9 (0.86-0.93) | 0.86 (0.8-0.9) | 0.87 (0.8-0.92) | 0.72 (0.64-0.8) |
| 160 | 0.9 | 0.91 (0.88-0.94) | 0.92 (0.9-0.94) | 0.9 (0.87-0.93) | 0.86 (0.82-0.89) | 0.88 (0.8-0.93) | 0.72 (0.66-0.78) |
| 320 | 0.9 | 0.91 (0.89-0.93) | 0.92 (0.91-0.93) | 0.9 (0.87-0.92) | 0.86 (0.83-0.88) | 0.89 (0.82-0.93) | 0.72 (0.68-0.76) |
| 640 | 0.9 | 0.91 (0.9-0.92) | 0.92 (0.91-0.93) | 0.9 (0.87-0.92) | 0.86 (0.84-0.88) | 0.89 (0.83-0.93) | 0.72 (0.69-0.75) |
| 1280 | 0.9 | 0.9 (0.9-0.92) | 0.92 (0.91-0.93) | 0.9 (0.87-0.92) | 0.86 (0.85-0.87) | 0.9 (0.85-0.92) | 0.72 (0.7-0.74) |

*Neither test within 5% of the true prevalence*

*Both tests within 5% of the true prevalence*

*Only test within 5% of the true prevalence*
