# Supplementary material for: Estimating prevalence and test accuracy in disease ecology: How Bayesian latent class analysis can boost or bias imperfect test results
Source: Ecol Evol. 2020 Jun 15;10(14):7221–32. doi: 10.1002/ece3.6448 (PMC7391344; doi:10.1002/ece3.6448)
Supplement: Supplementary file 11 — Supplementary Material [file ECE3-10-7221-s011.pdf]

Estimating prevalence and test accuracy in disease ecology: how Bayesian latent class analysis can boost or bias imperfect test results - *Research Code*

Single JAGS model run for estimates using California sea lion data:

1) Obtain binary test results for PCR, MAT and Serum Chemistry

2) Count the frequency of observations (total) and set up initial conditions for jags

3) Run the jags model and obtain estimates for prevalence, test sensitivity (s) & test specificity (x)

Note - Must have the jags file ("Supp_JAGSModel.txt") in the same folder as this script

California Sea Lion BLCA Example

```
#### IMPORT LIBRARIES ####
#library(R2jags)

#Set Working Directory (ensure JAGS file is in this folder)
setwd("~/Desktop/JAGS")




##################################################
####      California sea lion data example    #####
##################################################

# SET UP BINARY TEST RESULTS
# Dataset was filtered, and binary test results for 3 diagnostic tests are shown below


### COUNT the individuals in each profile (a-h) in your data
##############################################################
# a-h represent all possible test result combinations,
# ranging from all negative (profile a) to all positive (profile h)

#a <- sum(data$MAT==0 & data$SerumChem==0 & data$PCR==0)    #212
#b <- sum(data$MAT==0 & data$SerumChem==1 & data$PCR==0)    #15
#c <- sum(data$MAT==1 & data$SerumChem==0 & data$PCR==0)    #4
#d <- sum(data$MAT==1 & data$SerumChem==1 & data$PCR==0)    #0
```

```r
#e <- sum(data$MAT==0 & data$SerumChem==0 & data$PCR==1)    #10
#f <- sum(data$MAT==0 & data$SerumChem==1 & data$PCR==1)    #14
#g <- sum(data$MAT==1 & data$SerumChem==0 & data$PCR==1)    #16
#h <- sum(data$MAT==1 & data$SerumChem==1 & data$PCR==1)    #22


## SAVE these counts as your frequency of observations
#freqobs <- c(a,b,c,d,e,f,g,h)
freqobs <- c(212, 15, 4, 0, 10, 14, 16, 22)


#################################################################
#############    THREE TEST MODEL ESTIMATE    #################
#################################################################

test1 <- c(0,0,1,1,0,0,1,1)   #MAT
test2 <- c(0,1,0,1,0,1,0,1)   #Serum chemistry
test3 <- c(0,0,0,0,1,1,1,1)   #PCR

total <- sum(freqobs)



################### SET INITIAL CONDITIONS & RUN THE MODEL ############################

# Set 3 starting points for the model to run from. Here we use a low (10%), medium (50%)
#and high (90%) prevalence, along with low/med/high sensitivity and specificity values
#NOTE: Here s=sensitivity and x=specificity (rather than 'Se' & 'Sp' in text & Figure S2)
#Test 1 specificity is fixed, and is therefore NA


inits <- list(list(prev=0.5, s=c(0.5, 0.5, 0.5), x=c(0.5,0.5,NA)),
              list(prev=0.9, s=c(0.9, 0.9, 0.9), x=c(0.9,0.9,NA)),
              list(prev=0.1, s=c(0.1, 0.1, 0.1), x=c(0.1,0.1,NA)))


##### Run the model  ####
jags.fit <- jags(data=list(test1=test1, test2=test2, test3=test3, freqobs=freqobs,total=total),
                 inits=inits, #initial conditions - set above
                 parameters.to.save=c("prev", "s", "x"), #prevalence, sensitivity & specificity
                 model.file= "Supp_JAGSModel.txt",
                 n.chains=length(inits),
                 n.iter=10000)


############  CSL MODEL OUTPUT FOR 3 TEST MODEL ###############
#Model estimated prevalence, sensitivity (s) and specificity (x) for each test

jags.fit$BUGSoutput$summary     #summary table of all values
```

Simulated Data Generation for Multiple Sample Sizes

```
###########################################################################################
#############    SIMULATED DATA Based on California sea lion example  #####################
###########################################################################################

## This code simulates diagnostic test results based on the prevalence (20%)
#and diagnostic test accuracy predicted by BLCA using our California sea lion data (above)

#Set sample sizes you want to test
pops <- c(20, 40, 80, 160, 320, 640, 1280)

#Set known prevalence and test sensitivities/specificities based on CSL output
#(obtained in previous chunk using jags.fit$BUGSoutput$summary)
prev <- 0.2
sen1 <- 0.65
sen2 <- 0.61
sen3 <- 0.96
spec1 <- 0.98
spec2 <- 0.93
spec3 <- 0.972 ##NOTE: In "Supp_JAGSModel.txt", make sure
#the known specificty for test 3 matches the value here


#Set number of simulated data samples for each sample size
nsims=1000




#Make a dataframe to store the test results for each simulation
df_tests_all_sims <- data.frame(matrix(ncol = 9, nrow = nsims))



##########################################################
#####  SIMULATED TEST RESULTS (FREQOBS) #################
##########################################################

for (yy in 1:7){  #7 is the number of sample sizes you are testing

  #Set the sample size to test
  pop=pops[yy]

###Set up data frames to hold simulated outputs

  #coin flips for test results
df <- data.frame(matrix(ncol = 3, nrow = pop))

#sample size + freq obs (a-h) for each sim
df_testresults <- data.frame(matrix(ncol = 9, nrow = nsims))
```

```
################################
###### RUN THE SIMULATION ######
################################

  for (ii in 1:nsims){

###### SIMULATED TEST RESULTS FOR INFECTED INDIVIDUALS ########

    #Sensitivity: infected individual test results
    for (i in 1:(pop*prev)){
      df$X1[i]=rbinom(1,1,sen1)
      df$X2[i]=rbinom(1,1,sen2)
      df$X3[i]=rbinom(1,1,sen3)
    }


###### SIMULATED TEST RESULTS FOR UNINFECTED INDIVIDUALS ########

#Specificity: uninfected individual test results
    for (i in (prev*pop+1):pop){
      df$X1[i]=rbinom(1,1,(1-spec1))
      df$X2[i]=rbinom(1,1,(1-spec2))
      df$X3[i]=rbinom(1,1,(1-spec3))
    }




##########  FREQUENCY OF TEST PROFILE OBSERVATIONS ########

#Sum the frequency of test result combos
    a <- sum(df$X1==0 & df$X2==0 & df$X3==0)
    b <- sum(df$X1==0 & df$X2==1 & df$X3==0)
    c <- sum(df$X1==1 & df$X2==0 & df$X3==0)
    d <- sum(df$X1==1 & df$X2==1 & df$X3==0)

    e <- sum(df$X1==0 & df$X2==0 & df$X3==1)
    f <- sum(df$X1==0 & df$X2==1 & df$X3==1)
    g <- sum(df$X1==1 & df$X2==0 & df$X3==1)
    h <- sum(df$X1==1 & df$X2==1 & df$X3==1)

    hh <- c(a,b,c,d,e,f,g,h,i)

#Store sample size and test results (frequency of observations: a-h) in dataframe
    df_testresults[ii,]<-c(pop, hh[1], hh[2], hh[3], hh[4], hh[5], hh[6], hh[7], hh[8])

}


#Add the simulation results for each sample size to the previous results
    #to produce a dataframe with results for all simulations at all sample sizes
if (yy==1) {
    df_tests_all_sims <- df_testresults
```

```r
  } else {
    df_tests_all_sims <- rbind(df_tests_all_sims, df_testresults)
  }


}


#Column names: Sample size and test result profiles (a-h) for each simulation
colnames(df_tests_all_sims) <- c("pop", "000", "010", "100", "110", "001", "010", "101", "111")
```

These simulated test results are the frequency of observation ("freqobs") data inputs for the JAGS model, which can then estimate the prevalence, sensitivity (tests 1-3) and specificity (tests 1-2) based on the test profiles from each simulation.
