# Supplementary material for: Estimating prevalence and test accuracy in disease ecology: How Bayesian latent class analysis can boost or bias imperfect test results
Source: Ecol Evol. 2020 Jun 15;10(14):7221–32. doi: 10.1002/ece3.6448 (PMC7391344; doi:10.1002/ece3.6448)
Supplement: Supplementary file 12 — Supplementary Material [file ECE3-10-7221-s012.docx]

**Figure S1: Sensitivity and specificity values for test 1 & test 2 (red points) that were used in conjunction with Test 3 (fixed arc points A-E) for data simulations.** (A) Original test 1 and test 2 values, corresponding to the simulation results in Figure 3. (B) Alternate test 1 and test 2 values, corresponding to the simulation results in Figure S3. (C) Alternate test 1 and test 2 values, corresponding to the simulation results in Figure S4.

**Figure S2: JAGS Model and probability functions for each test result profile (a-h).**

(A) Conditional independence model used in JAGS, assuming that each individual’s test results are independent given true disease status. (B) Explanation of the JAGS model. (C) Probabilities for the number of individuals expected in each test profile category for a given sample size (𝐧) and prevalence (𝛑).

**Figure S3: Parameter estimates at three true prevalence levels (10%, 50% & 90%) using the first alternate scenario test 1 (Se_1_=0.9 and Sp_1_=0.7) and test 2 (Se_2_=0.7 and Sp_2_=0.9).** *Left:* Median prevalence estimates and 95% credible intervals (CrI) are shown for points A-E at a true prevalence of 10% (A), 50% (B) and 90% (C), with true prevalence shown as dashed black lines (y-axes scaled equally). *Right:* Residuals for all parameter estimates (prevalence, sensitivities for tests 1-3, specificities for tests 1 & 2) using simulated samples (n=1000) generated with fixed arc point C (test 3 fixed Se=95% and Sp=95%), with zero shown as dashed red line.

**Figure S4: Parameter estimates at three true prevalence levels (10%, 50% & 90%) using the second alternate scenario for test 1 (Se_1_=0.8 and Sp_1_=0.6) and test 2 (Se_2_=0.5 and Sp_2_=0.9).** *Left:* Median prevalence estimates and 95% credible intervals (CrI) are shown for points A-E at a true prevalence of 10% (A), 50% (B) and 90% (C), with true prevalence shown as dashed black lines (y-axes scaled equally). *Right:* Residuals for all parameter estimates (prevalence, sensitivities for tests 1-3, specificities for tests 1 & 2) using simulated samples (n=1000) generated with fixed arc point C (test 3 fixed Se=95% and Sp=95%), with zero shown as dashed red line.

**Figure S5:** Prior distributions used to estimate prevalence. Panel A shows an uninformed prior (uniform distribution on [0,1]). Panels B-D show informed prevalence priors (prevalence ~ beta(2,9), beta(9,9) and beta(9,2) for suspected low, medium and high prevalence respectively).

**Figure S6: Comparison of simulation results using uninformative prevalence priors versus informed priors with suspected low, medium and high prevalence.** Each row represents results for a known true prevalence: 10% (top), 50% (middle) or 90% (bottom). Each pair of columns represents the prevalence prior: uninformed (left), low (left middle), medium (right middle) and high (right)*.* Within each column pair, the left column represents the median prevalence by sample size (true prevalence shown as dashed black line; y-axes scaled equally), and the right column illustrates the residuals for all estimated parameters (zero shown as dashed red line).

**Figure S7: Simulation results from model estimates using California sea lion (CSL) data.** (A) Diagram showing the location of the CSL test estimates (shown in red) relative to tests previously reported in the literature (shown in black). (B) Model estimates of prevalence by sample size, using simulated data based on CSL tests, when true prevalence = 20% (dashed black line). (C) Residuals for all parameter estimates, with the true values at zero (dashed red line).

**Table S1:** Median prevalence estimates with 95% CrI for all arc points (A-E), shown at increasing sample sizes (n=20, 40, 80, 160, 320, 640, 1280), when true prevalence is 10% (top), 50% (middle) and 90% (bottom).

**Table S2: Comparison of prevalence estimates using BLCA to those obtained with a single diagnostic test.** BLCA results (using fixed point A) versus test A alone (left two columns), BLCA results (using fixed point C) versus test C alone (middle two columns), and BLCA results (using fixed point E) versus test E alone (right two columns). Results are shown for all sample sizes when true prevalence is 10% (S2A), 50% (S2B) and 90% (S2C). Sensitivity and specificity for tests A, C and E were Se_3_=1.0/Sp_3_=0.8, Se_3_=0.95/Sp_3_=0.95 and Se_3_=0.8/Sp_3_=1.0 respectively.
